# Complex Limb Injuries: Limb Salvage Versus Amputation—A Mini Review and Meta-Analysis

**DOI:** 10.1155/aort/2884802

**Published:** 2025-01-30

**Authors:** Athanasios Serlis, Panagiotis Sgardelis, Themistoklis Vampertzis, Konstantinos Rizavas, Panagiotis Poulios, Georgios Konstantopoulos

**Affiliations:** ^1^Trauma & Orthopaedic Department, Cambridgeshire and Peterborough NHS Foundation Trust, Cambridge, UK; ^2^Trauma & Orthopaedic Department, Norfolk and Norwich University Hospital, Norwich, UK; ^3^Trauma & Orthopaedic Department, University Hospital Southampton NHS Foundation Trust, Southampton, UK; ^4^Trauma & Orthopaedic Department, Barts Health NHS Trust, London, UK; ^5^Trauma & Orthopaedic Department, Royal National Orthopaedic Hospital, London, UK; ^6^Trauma & Orthopaedic Department, West Suffolk NHS Foundation Trust, Bury St Edmunds, UK

## Abstract

**Introduction:** The management of complex limb injuries can be very challenging, and it demands a multidisciplinary approach to treatment. Amputation and limb reconstruction are the two options that clinicians must choose. This study aims to comprehensively synthesize existing tools and resources from the literature that can assist clinicians in the decision-making process.

**Evaluation:** The initial resuscitation and the prehospital care are the first important steps in the management of these injuries, while the immediate transfer to trauma centers is recommended for complex cases. After the stabilization of the patient, a thorough clinical examination of the limb is necessary with emphasis on the degree of soft tissue damage. Blunt trauma in the lower limb is associated with a higher risk of early amputation. Polytrauma patients with complex limb injuries require a holistic approach, with Damage Control Orthopedics (DCO) principles. Traumatic bleeding significantly increases mortality rates, necessitating prompt control using pressure bandages or tourniquets. Computed tomography angiography (CTA) is necessary in order to assess the viability of the limb.

**Management:** Scoring systems can be used as a tool in the management of complex lower and upper limb injuries. Mangled Extremity Severity Score (MESS) calculates ischemia, shock, bone and soft tissue damage, and patient characteristics. The Narakawa Index (NISSSA score) constitutes an alteration of MESS with the implementation of a nerve injury element. The Musculoskeletal Score for Severity of Injury (MESI score) estimates the risk of limb amputation by evaluating injury, neurovascular damage, type of fracture, patient characteristics, and the period from the occurrence of trauma to the definitive treatment. Further interventions and patient preferences should be incorporated into the decision-making process.

**Outcome:** The outcomes of limb salvage versus amputation for complex limb injuries encompass various factors, including patient's preinjury health status, psychological well-being, functional outcomes, and economic impact. While some studies suggest better psychological outcomes with limb reconstruction and others find similar functional outcomes between the two approaches, economic considerations play a significant role in decision-making.

**Conclusion:** Managing complex limb injuries effectively necessitates a comprehensive approach involving thorough assessment, multidisciplinary collaboration, and patient-centered care. Given the diverse factors influencing management and long-term outcomes, it is crucial to integrate medical expertise with patient preferences and expectations.

## 1. Introduction

Over the last decades, the development of well-organized trauma systems has provided significant advantages in the treatment of the mangled extremity. The available literature provides useful information with regard to the management of complex limb injuries; however, the severity of bone, vascular, and soft tissue damage makes their initial assessment and management extremely challenging. Therefore, a multidisciplinary team approach, composed of orthopedic, plastic, and vascular surgeons, is required to help clinicians identify the most appropriate treatment plan by considering patient factors and preferences efficiently.

Clinicians must consider multiple variables to determine whether the advantages of limb reconstruction or amputation outweigh the disadvantages in each case. The decision-making process is determined by the efficacy of the emergency services, the type of trauma unit, the experience of the clinicians, and the factors of the patient. Also, mangled extremity is the result of high energy trauma and is usually associated with life-threatening head, abdominal, or pelvic injuries which inevitably affect the final outcome.

Limb amputation is a challenging and unpleasant process for both patients and clinicians due to its enormous effects on their quality of life and psychology. However, preservation of life remains the primary concern, and surgeons must prevent further complications of the severely injured patient that could potentially emerge from limb salvage procedures.

## 2. Evaluation

Complex limb injuries could distract clinicians' attention from associated fatal injuries that can lead to patient's rapid deterioration. Although a detailed clinical examination is essential in the assessment of the mangled limb, particular attention should be paid to the initial resuscitation and management of life-threatening injuries based on advanced trauma life support principles. More specifically, the coordination and cooperation of emergency services and the hospital setting are of paramount importance to mediate the catastrophic effects of these injuries.

Firstly, prehospital services constitute the pillar of initial management and a crucial determinant of the final outcome. They initiate life-saving interventions, such as controlling bleeding and immobilizing the affected area, which are vital to preserve limb function. In addition, prompt transportation to appropriate medical facilities ensures that patients receive timely surgical care, improving overall outcomes.

The National Institute for Health and Care Excellence guidelines recommend immobilization of the affected limb and early intravenous antibiotic administration in the prehospital setting [1]. Lack et al. supported that antibiotic administration within the first hour after injury reduces deep infection rates in complex limb injuries, but these results should be treated with caution due to the insufficient follow-up period of the study [2].

Complex lower and upper extremity injuries should be immediately transferred to trauma centers that can provide joint orthoplastic care [1]. Naique et al. showed that the treatment of complex extremity injuries in major trauma centers is associated with fewer delayed amputations compared to district general hospitals [3]. This study has statistically significant results, but the lack of documentation of soft tissue damage and the small sample size creates concerns about its validity.

The initial stabilization of the patient should be followed by a thorough clinical assessment of the mangled extremity. A detailed clinical examination of the upper or lower limb is essential to classify soft tissue and skin damage, identify bone injuries, and assess neurovascular status. Hafez et al. showed that patients with neurovascular deficit or signs of compartment syndrome in the initial assessment had an increased risk of amputation [4]. Despite the large sample size, this study reviewed only injuries in the lower extremities injuries; thus, its results cannot be generalized to the population at large.

Furthermore, the degree of soft tissue damage is a crucial determinant of the final outcome. Faris et al. supported that the severity of soft tissue damage is associated with an increased risk of early amputation. The authors provided statistically significant results through an adequate number of patients, but there was a lack of standardization of fixation type, and the study focused only on injuries to the lower extremities [5].

In addition, blunt trauma to the lower limb is associated with a high risk of amputation. Alberty et al. reported an amputation rate of 40% in cases with blunt trauma, while Mc Cabe et al. showed that 21% of patients who suffered blunt force trauma required amputation [6, 7]. Despite the limited number of cases, both studies have highlighted that the role of clinical suspicion is essential in the initial evaluation of blunt trauma and could prevent delayed diagnosis and unnecessary amputations in the mangled extremity.

Polytrauma patients usually suffer from multiple injuries, including complex lower or upper limb trauma. The principles of Damage Control Orthopedics (DCO) should be applied in the assessment and management of these patients to achieve the best outcome and reduce systemic complications. Roberts et al. mentioned that bleeding control and patient stabilization offer better results compared to fracture repair and reconstruction in critically ill patients [8]. Harwood et al. supported that DCO in unstable patients with femoral fractures could minimize the inflammatory response and reduce life-threatening complications. However, the authors reviewed only the efficiency of DCO in femoral fractures; therefore, the results cannot be applied to all complex limb injuries [9].

Traumatic bleeding significantly increases mortality rates significantly. Therefore, control of external bleeding, with pressure bandages or use of tourniquets in the emergency setting, could be life-saving. More specifically, external bleeding causes 10% of total deaths on the battlefield [10]. However, a study by Kragh et al. revealed that tourniquets were associated with high rates of amputation and fasciotomy in complex limb injuries. The large number of patients reinforces the validity of this study, but the results need to be treated with caution as the included population is restricted to the combat zone [11].

Furthermore, imaging is one of the main tools that usually help clinicians identify bone and vascular damage in the mangled extremity. The initial evaluation of complex limb injuries usually includes computed tomography angiography (CTA) in order for surgeons to be able to assess the viability of the limb. Fung Kon Jin et al. reported that CT scans in severely injured patients could last up to 19 min. Considering that delays could lead to rapid deterioration of the patient, clinicians should carefully measure if additional imaging or prompt intervention would be more beneficial [12].

## 3. Management

Several authors have attempted to implement scoring systems that could be used as a tool in the management of complex lower and upper limb injuries. Johansen et al. described MESS which predicted the need for amputation in patients scoring above 7, calculating ischemia, shock, bone and soft tissue damage, and patient characteristics [13]. Badole et al. showed that MESS could estimate the risk of amputation with sensitivity and specificity of 91% and 98%, respectively, in 58 patients with complex limb injuries [14]. However, this study is characterized by a significant risk of bias due to its retrospective design. On the other hand, Menakuru et al. demonstrated significantly lower sensitivity and specificity rates through a study of 148 patients [15]. Brown et al. showed that MESS was inaccurate as a predictive factor of the need for amputation [16].

The NISSSA score constitutes an alteration of MESS with the implementation of a nerve injury element. Bosse et al. showed that plantar sensation was restored in more than half of the patients that underwent limb reconstruction procedure [17]. Therefore, the absence of plantar sensation as a component of this scoring system was criticized as an imprecise predictive factor. The MESI score estimates the risk of limb amputation by evaluating injury, neurovascular damage, type of fracture, and patient characteristics, and the period from the occurrence of trauma to the definitive treatment [18].

**Figure 1:**
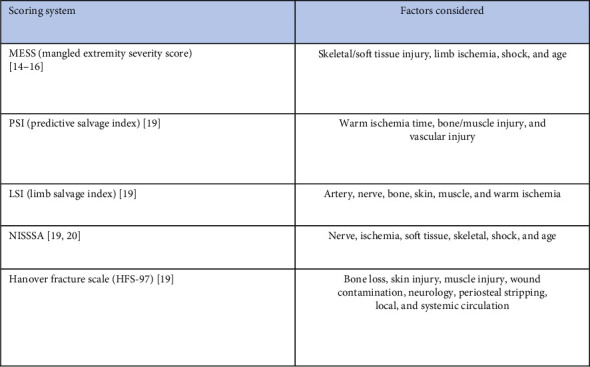
Mangled extremity scoring systems.

However, current evidence in the literature shows that the clinical benefit of the above scores is limited. More specifically, Bosse et al. evaluated the use of five injury severity scores (MESS, LSI, PSI, NISSSA, and HFS-97), highlighting that high scores were not necessarily associated with an increased risk of amputation [19], (Figure 1). The Lower Extremity Assessment Project (LEAP) also questioned the validity of the injury severity scores by prospectively reviewing 601 patients with complex injuries. The LEAP study aimed to identify the profiles of individuals who experienced these injuries, examine their environmental factors, analyze the physical attributes of the injuries, assess the secondary medical and mental health issues that emerged from the injuries and their treatment, evaluate their long-term functional outcomes, and explore their overall health status. However, the LEAP study was located in the United States, and there was no randomization of the treatment options; therefore, its reliability should be thoroughly evaluated [20].

Furthermore, complications constitute an inevitable element of the decision-making process. Clinicians should take into account the impact that further interventions could have on patient psychology and well-being. Harris et al. supported that lower extremity injuries are related to delayed bone healing, wound infection, and chronic osteomyelitis. The authors highlighted that revision surgery was required in approximately 5% of the total patients who underwent a primary amputation, while in the group of late amputation, the complication rate peaked at 85% [21]. Bondurant et al. supported that primary amputation is associated with fewer operations and hospitalization days compared to amputations after limb salvage procedures. Despite the lack of standardization in the type of fixation and the inaccurate definition of muscle necrosis, the large number of patients included in this study increases its validity [22].

Limb amputation is a life changing procedure for both patients and their families, while limb reconstruction is associated with multiple complications. Patients should be incorporated into the decision-making process to be aware of the risks and benefits of each procedure. Aravind et al. showed that patients who suffered complex lower limb injuries over 10 years felt that they were not adequately involved in the treatment choice process (limb salvage versus amputation) [23]. This study also highlighted that medical professionals play a crucial role, mainly because patients were unconscious or drowsy during the critical period. Although this study has a limited number of participants (20) and is characterized by selection bias, its results emphasize that patients' preferences should be an indispensable part of the whole process.

Finally, the level of amputation is a significant component of the management process and is usually defined by the preoperative status of the patient. Spence et al. demonstrated that above-knee amputation is preferable in old patients with multiple comorbidities, while the functional results of patients with below-knee amputation are similar to successful limb reconstruction procedures. The statistically significant results and the large number of patients increase the reliability of the study, despite the retrospective design and the risk of selection bias.

### 3.1. Outcomes of Limb Salvage Versus Amputation

Regardless of the treatment choice, there are specific factors that constitute significant indicators of the final outcome. Whether limb salvage or amputation provides better results is not a straightforward answer, and careful evaluation of the patient's preinjury status is required.

The Sickness Impact Profile (SIP) score in the LEAP study, which demonstrates the health status of the patients through 12 categories, was similar between the limb reconstruction and amputation mentioned that low scores were related to low sociofinancial status, high rates of rehospitalization, and inadequate social support, while low self-efficacy was an indicator of unsatisfactory results. Despite the lack of randomization, the LEAP study showed through a sufficient follow-up period that the background of each patient defines the final outcome. Akula et al. conducted a meta-analysis that showed better psychological results in the limb reconstruction group compared to the amputees [24]. Authors used a comprehensive literature search and statistical methods to prove their results, but the conclusions should be treated with caution due to the significant risk of bias.

Furthermore, the long-term functional outcome is the main objective of clinicians and patients; thus, the choice of treatment should improve quality of life as much as possible. However, Busse et al. showed that both limb salvage and amputation offered similar functional outcomes in 7 years [25]. Furthermore, the authors reported that disability rates fluctuated between 40% and 50% between the two groups; therefore, the choice of treatment did not affect the overall outcome. The sufficient follow-up period is the main strength of this study, but the lack of randomization and blinding decreases the significance of the results. Georgiadis et al. reviewed the functional result of patients who underwent reconstruction or amputation after a Grade III-B or III-C tibial fracture. The authors showed that the patients in the limb salvage group had worse functional and psychological status, lower personal expectations, and higher hospital charges compared to amputees. The generalizability of the results creates concerns about the study findings despite statistically significant data and a satisfactory follow-up period [26]. Doukas et al. reported that the functional outcome was significantly better in the amputee group after military extremity trauma. However, the interpretation of the results should be made with caution due to the risk of selection bias [27].

Finally, complex limb injuries have a significant economic impact on individuals and the healthcare system. Treatment choice is not affected by the latter, but patients usually have to bear the financial burden of rehabilitation. More specifically, Mackenzie et al. supported that the total cost for amputee treatment and rehabilitation is three times greater than that of the limb salvage group [28]. The authors supported that the limb reconstruction procedure reduces lifetime expenses. However, this study was limited to eight specific trauma centers and its results could not be generalized.

## 4. Conclusion

**Figure 2:**
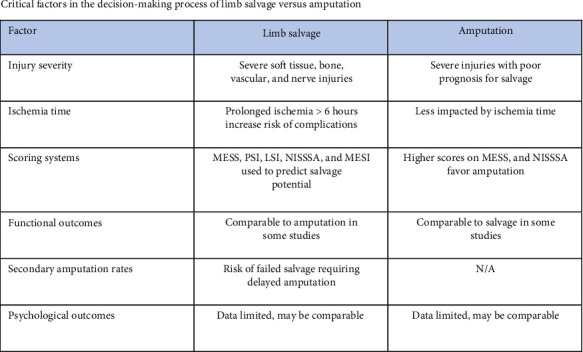
Critical factors in the decision-making process of limb salvage versus amputation.

Complex limb injuries require thorough assessment, multidisciplinary team participation, and patient-centered care to achieve the best outcome. Multiple components affect the management of these injuries and long-term outcomes of patients; therefore, the combination of medical expertise and patient preferences and expectations is essential to achieve the ideal outcome and minimize possible complications (Figure 2).

**Figure 3:**
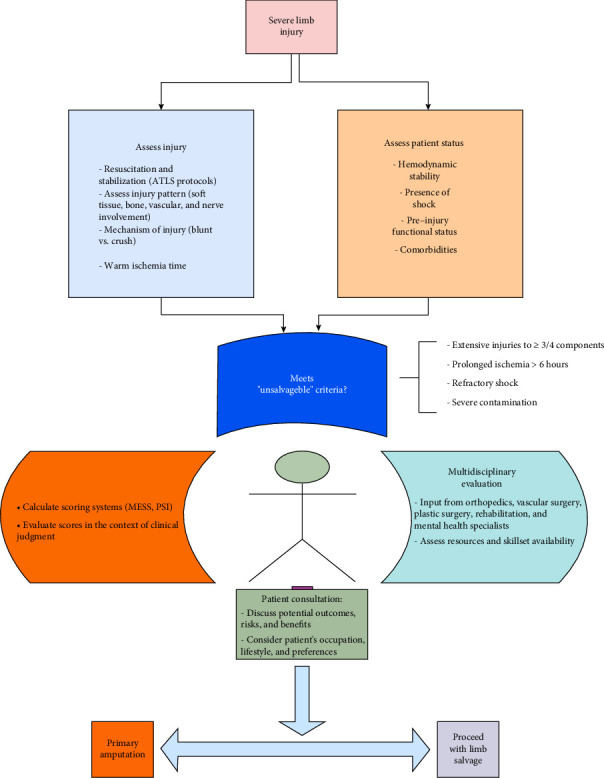
Decision-making flowchart.

The key factors influencing the decision-making process for limb salvage versus amputation include the severity and extent of injury, particularly to soft tissue, bone, vascular, and nerve systems, as well as the duration of ischemia, with prolonged ischemia (> 6 h) increasing the likelihood of amputation. The use of scoring systems such as MESS, PSI, and NISSSA helps predict salvage potential, while data on functional outcomes and secondary amputation rates postsalvage attempts provide further insights into treatment efficacy (Figure 3). In addition, psychological factors such as quality of life, depression, and PTSD play a crucial role in assessing overall outcomes and patient well-being after either intervention.

## Data Availability

The data used to support the findings of this study are included within the article.
